# Dentoalveolar effects of skeletally anchored extrusion arch in anterior open bite patients: A prospective clinical trial

**DOI:** 10.1590/2177-6709.28.6.e2323110.oar

**Published:** 2024-01-05

**Authors:** Noheir Samir ELSHAL, Mohammad Hasan MOHAMMAD, Marwa Ali TAWFIK, Maher Abd El-Salam FOUDA

**Affiliations:** 1 Mansoura University, Faculty of Dentistry, Department of Orthodontics (Mansoura, Egypt).

**Keywords:** Extrusion arch, Open bite, Digital models, Cephalogram, Extrusion

## Abstract

**Objective:**

The purpose of this prospective clinical trial was to explore the dental and soft tissue changes accompanying the use of skeletally anchored nickel-titanium (NiTi) extrusion arch in the correction of anterior open bite (AOB).

**Material and Methods:**

Twenty female patients with a mean age of 16.5 ± 1.5 years and a mean dentoalveolar AOB of 2.38±0.7 mm participated in this study. All patients were treated with an maxillary 0.017×0.025-in NiTi extrusion arch, with the aid of miniscrews inserted between the maxillary second premolars and first molars bilaterally, to act as indirect anchorage. Three-dimensional digital models and lateral cephalometric radiographs were taken just before the insertion of the extrusion arch (T0) and after 10 months (T1). Paired-sample *t*-tests were used in analyzing the data, to evaluate the changes after treatment (T1-T0). A significance level of *p* < 0.05 was used.

**Results:**

AOB was successfully closed in all patients, with a 4.35 ± 0.61 mm increase in the overbite. Maxillary incisors significantly extruded (2.52 ± 1.02 mm) and significantly reclined (5.78 ± 0.77°), with a resultant decrease in the overjet of 1.58 ± 0.5mm. A significant intrusion of maxillary first molars with no change in their inclination was observed. The upper lip showed a significant retraction tendency to the E-plane, and a significant increase in the nasolabial angle was observed.

**Conclusion:**

The skeletally anchored NiTi extrusion arch was an effective technique in treating AOB, with no adverse effects on the molars.

## RESUMO

**Objetivo:**

O objetivo deste ensaio clínico prospectivo foi avaliar as alterações dentárias e em tecidos moles que acompanham o uso de arco de extrusão de níquel-titânio (NiTi) com ancoragem esquelético na correção da mordida aberta anterior (MAA).

**Material e Métodos:**

Participaram deste estudo 20 pacientes do sexo feminino com idade média de 16,5 ± 1,5 anos e MAA dentoalveolar (média de 2,38 ± 0,7 mm). Todos os pacientes foram tratados com arco de extrusão de NiTi 0,017×0,025” superior, com auxílio de mini-implantes inseridos entre os segundos pré-molares e primeiros molares superiores bilateralmente, para atuar como ancoragem indireta. Modelos digitais tridimensionais e radiografias cefalométricas laterais foram realizados imediatamente antes da inserção do arco de extrusão (T0) e após 10 meses (T1). Testes t para amostras pareadas foram utilizados na análise dos dados, para avaliar as alterações após o tratamento (T1-T0). Um nível de significância de *p*< 0,05 foi usado.

**Resultados:**

A MAA foi fechada com sucesso em todos os pacientes, com aumento de 4,35 ± 0,61 mm na sobremordida. Os incisivos superiores foram significativamente extruídos (2,52 ± 1,02 mm) e significativamente reclinados (5,78 ± 0,77°), com uma consequente diminuição na sobressaliência de 1,58 ± 0,5 mm. Foi observada intrusão significativa dos primeiros molares superiores, sem alteração na sua inclinação. O lábio superior apresentou tendência significativa de retração em relação ao plano E, e foi observado aumento significativo do ângulo nasolabial.

**Conclusão:**

O arco de extrusão de NiTi esquelético foi uma técnica eficaz no tratamento da MAA, sem efeitos adversos nos molares.

## INTRODUCTION

Open bite is a type of malocclusion frequently encountered in dental practice. Well-planned treatment of an open bite is the only key to a stable and successful treatment. Anterior open bite (AOB) is defined as the lack of the positive overlap of the maxillary incisors over the mandibular ones.^1^

AOB is generally classified into two types: skeletal and dentoalveolar open bite.^1^ ^,^ ^2^ Based on its severity, AOB of 0-2mm is considered as mild; 3-4mm, as moderate; and greater than 4mm, as severe.^3^ As a result of different treatment modalities, diagnosis is important.^4^ ^,^ ^5^ Studies have shown that skeletal open bite is often associated with excessive vertical growth of the alveolar bone of the molars, with occlusal surfaces diverging anteriorly from the molars.^6^ On the other hand, dentoalveolar AOB is primarily due to a reduction in the vertical height of the alveolar bone of the incisors, with occlusal surfaces diverging anteriorly from the first premolar.^7^ ^,^ ^8^

Generally, dentoalveolar AOB can be treated with Orthodontics alone, whereas true skeletal open bites require a combination of Orthodontics and Orthognathic Surgery. Orthodontic treatment of dentoalveolar AOB includes either anterior teeth extrusion or/posterior teeth intrusion. 

 Different treatment modalities have been developed to extrude the anterior teeth, such as multiloop archwires associated with vertical elastics,^9^ and the use of upper accentuated and lower reverse-curve archwires with intermaxillary elastics.^10^ Despite excellent results, most of these treatments need patient compliance and may cause discomfort. Therefore, fixed device techniques that do not depend on patient cooperation, such as extrusion arches, are increasingly being adopted.^11^ ^-^ ^13^

The TMA extrusion arch is considered a one-couple force system that produces an extrusive force on the anterior teeth, allowing the AOB correction. Its activation depends on a V-shaped bend that is located 1-3 mm anterior to the molar tube, exerting an extrusive force of 40-60 g on the anterior teeth.^11^ ^,^ ^14^ However, mesial tipping of the posterior teeth occurs due to the counter-clockwise couple developed by the one-couple force system. So, Uribe et al.^7^ suggested using a Connecticut intrusion arch fabricated of nickel-titanium, by inverting it. It delivers a lower force of 30-40 g, which aids in minimizing the unfavorable effects on maxillary molars. 

No research studies have reported the dental and soft tissue effects of using a NiTi extrusion arch in the treatment of AOB. Therefore, the purpose of the present study was to evaluate the dental and soft tissue changes associated with the use of a skeletally anchored NiTi extrusion arch in treating patients with dentoalveolar open bite. The null hypothesis was that a skeletally anchored NiTi extrusion arch would not cause any dental or soft tissue changes while correcting the AOB. 

## MATERIAL AND METHODS

### TRIAL DESIGN

This study was approved by the Dental Research Ethics Committee, Faculty of Dentistry, Mansoura University (M05060421) as a prospective clinical trial. The study was registered at clinicaltrials.gov (NCT05492864). This study was conducted from May 2021 to December 2022. Written consent was obtained from patients and guardians after they were briefed on the study and its implications.

### SAMPLE SIZE CALCULATION

This study planned to have a power of 98%, based on the previous study by de Brito Vasconcelos et al.^15^ A sample size of 15 patients was calculated to detect a mean of 1.9mm paired differences in overbite, and the estimated standard deviation of the difference was 1.7 mm, with an alpha level of 0.05, using a two-tailed paired *t*-test. To compensate for the dropout and reject the null hypothesis, the sample size was increased to 20 patients.

### PARTICIPANTS COLLECTION

**Figure 1:**
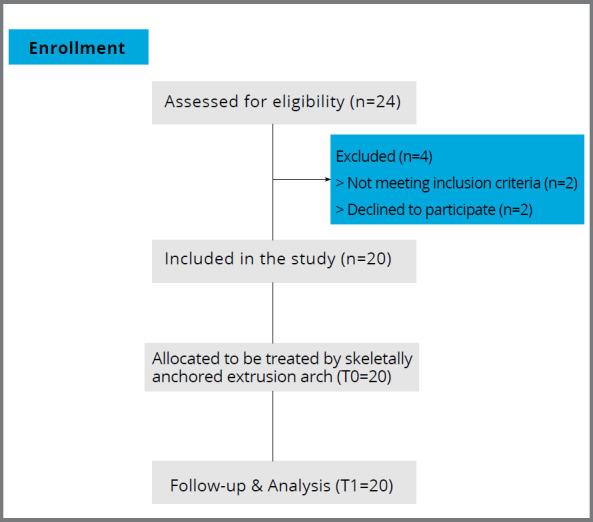
Flow chart of the patients through the study.

A total of 24 patients were assessed for eligibility, according to the following inclusion criteria: adolescent patients (age ≥ 15 years) with full permanent dentition, except for the third molars, skeletal Class I and Angle Class I relation, normal or minimally increased facial height, mild to moderate open bite (AOB ≥ 2mm) with no crowding, and low lip line. Those who had a skeletal open bite, excessive gingival exposure on smiling, trauma to the maxillary incisors, and those who needed extraction treatment due to excessive crowding and posterior crossbite were excluded from the study. Twenty patients met the inclusion criteria and consented to join the study (Fig 1). The following pretreatment records were taken for all candidates: lateral cephalometric and panoramic radiographs, study models, intraoral and extraoral photographs. The same operator treated all the candidates in the Department of Orthodontics at Mansoura University.

### INTERVENTION AND EXTRUSION MECHANICS

**Figure 2:**
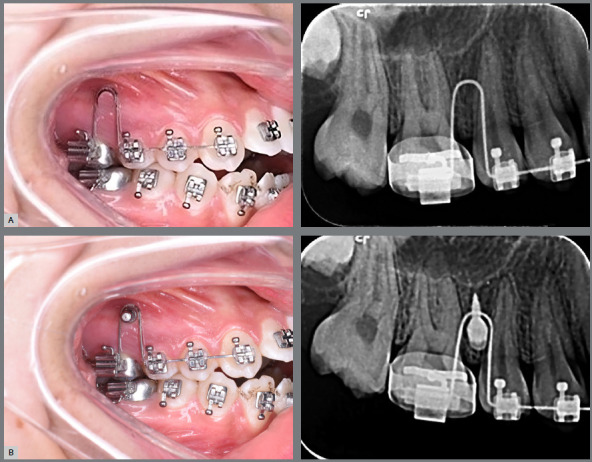
Intraoral photographs and periapical radiographs with the location guide before insertion of the miniscrew (A) and after insertion of the miniscrew (B).

Triple and double tube bands with suitable size were banded to the maxillary and mandibular first permanent molars respectively. Then, both arches were aligned until reaching the 0.017×0.025-in sectional stainless steel (SS) archwire for the maxillary arch and continuous archwire for the mandibular arch, using conventional brackets (0.022-in slot, Roth prescription, Morelli Ortodontia, Sorocaba/SP, Brazil). A location guide was made using 0.016×0.22-in SS wire, to detect the site of insertion of the orthodontic miniscrew into the bone. Then, a periapical radiograph was taken using a film holder, to determine the guide position relative to the adjacent teeth roots. A self-drilling titanium alloy miniscrew with a mushroom-shaped head type (1.6 mm in diameter and 8 mm in length, Dentaurum Inc, USA) was inserted through the attached gingiva into the buccal alveolar bone on each side, between the roots of maxillary second premolars and first permanent molars. Then, another periapical radiograph was taken, after the insertion of the miniscrew, to ensure that it was inserted in the correct position (Fig 2). They were tied immediately to the maxillary first permanent molar bands, on the right and left sides, using ligature wire (Remanium^®^ preformed ligature, 0.25 mm), to act as indirect anchorage. A buccally positioned 0.017×0.025-in rigid sectional SS archwire from the first molar to the canine was added for anchorage reinforcement. 

**Figure 3:**
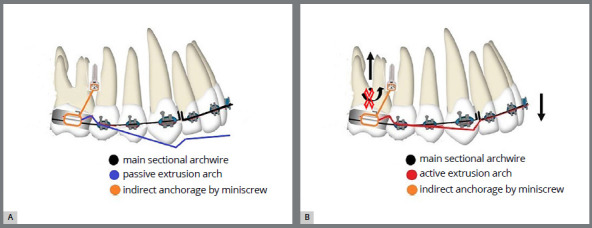
Schematic diagrams showing the maxillary NiTi skeletally anchored extrusion arch mechanics: A) passive extrusion arch, B) active extrusion arch.

**Figure 4:**
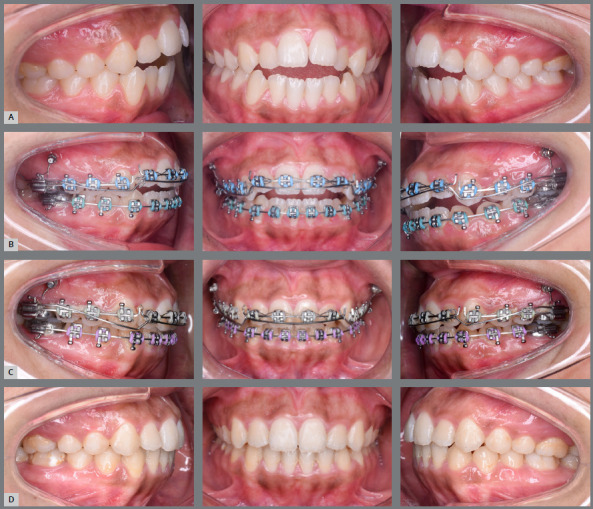
A) Pre-treatment intraoral photographs of a patient with AOB. B) Just after insertion of the skeletally anchored extrusion arch (T0). C) After 10 months (T1), with correction of the AOB. D) Post-treatment intraoral photographs.

**Figure 5:**
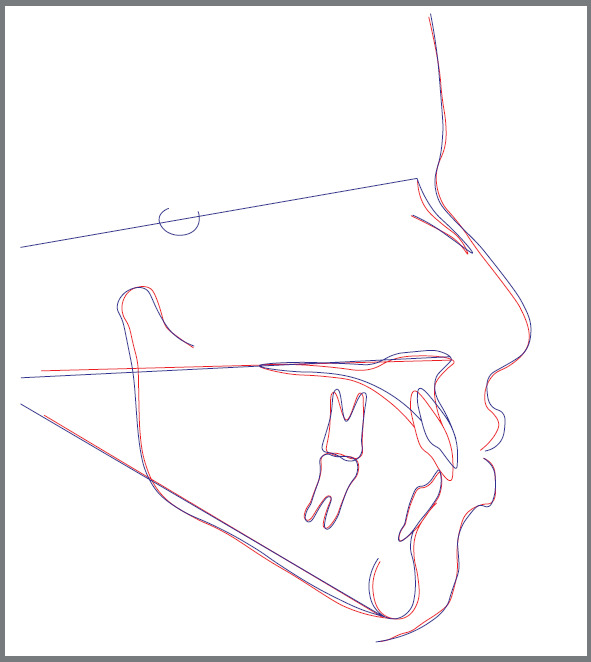
Cephalometric tracings superimposition: at T0 (blue line) and results at T1 (red line).

The anterior teeth were ligated and a sectional 0.017×0.025-in SS archwire was inserted. Extrusion was done by inverting the 0.017 × 0.025-in NiTi Connecticut intrusion archwire (Ortho Organizers, Inc.) as an overlay archwire, and inserting it in the auxiliary tubes of the molar bands (Fig 3). The NiTi extrusion arch had a V-shaped bend located 1-3mm anterior to the tube of the maxillary molar band, then a force gauge was used to deliver 30-40 g vertical extrusive force, as recommended by Uribe et al.^7^ Then, the extrusive arch was tied distal to the maxillary lateral incisor bracket, over the anterior wire, using a metal ligature, to provide the proper force application point and the correct moment-to-force ratio, with the use of a one couple force system. Follow-up visits were scheduled every month, and the extrusion arch was maintained for 10 months (follow-up period) (Figs 4, 5).

The patients were provided with an oral hygiene kit containing toothbrush and toothpaste (Colgate^®^ Palmolive Egypt), mouthwash (Oral-B^®^, Egypt), and dental floss. They were instructed to brush their teeth at least twice a day and floss once a day. Patients were notified to request an emergency visit if they had problems with their orthodontic appliance. 

After ten months of follow-up, lingual spurs (Ortho Organizer, Inc, Carlsbad, USA) were bonded with composite resin on the palatal surfaces of the upper central incisors, to reeducate the tongue position and to aid in the stability of the treatment. Treatment was finished using settling elastics (1/4-in medium, Ortho Technology, Tampa, Fla, USA) in the posterior segment, to settle the occlusion. At the end of orthodontic treatment, debonding was performed and the following retention protocol was applied to all patients: upper Hawley retainer with tongue guard, and lower fixed retainer.

### THREE-DIMENSIONAL MODEL ANALYSIS

**Figure 6:**
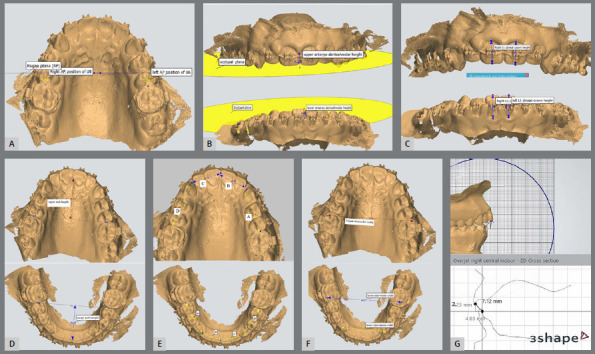
Digital model measurements. **A**) AP U6 (the linear distance between the rugae plane (RP) and the maxillary first molar mesial surface) - three points were used to determine the RP: the medial point of the left and right third rugae of the palate, and a point just opposite to the last point on the midpalatine raphe. **B**) UADH and LADH (the vertical distance between the occlusal plane and a point at the alveolar process between the central incisors, from the frontal view) - three points were used to determine the occlusal plane: mesiobuccal cusp tip of the right and left first permanent molars, and cusp tip of the right first premolar. **C**) U1 L and L1 L (the vertical distance between the incisal edge and gingival margin of the central incisor along its labial surface). **D**) UAL and LAL (the perpendicular linear distance from the contact point between the central incisors to a line connecting the permanent first molars mesial surfaces). **E**) UAP and LAP - the sum of four segments: from the contact point between the central incisors to the mesial contact point of the canine, and then to the mesial contact of the permanent first molar, measured on the right and left sides. **F**) U6-6 and L6-6 - the linear distance between the right and left mesiobuccal cusp tip of the first permanent molar. **G**) Overbite and overjet.

Three-dimensional models, obtained using an intraoral scanner (Heron™ IOS), were analyzed by the 3Shape Ortho Analyzer^TM^ software. The following variables were measured: overbite, overjet, the upper first molar antero-posterior position (AP U6), upper and lower anterior dentoalveolar height (UADH and LADH), upper and lower central incisor clinical crown length (U1 L and L1 L), upper and lower arch length (UAL and LAL) and perimeter (UAP and LAP), and upper and lower intermolar width (U6-6 and L6-6) (Fig 6). The variables were analyzed two times for all patients: just before the insertion of the extrusion arch (T0), and after 10 months (T1).

### LATERAL CEPHALOMETRIC ANALYSIS

**Figure 7:**
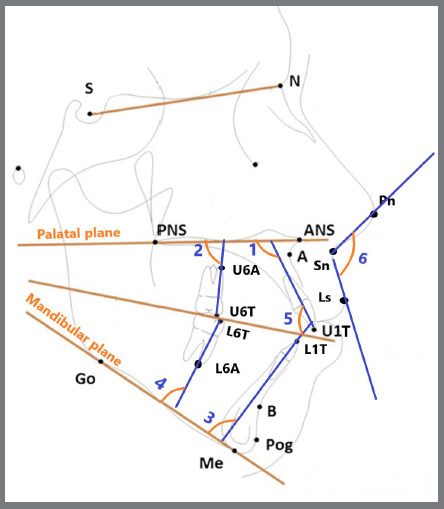
Schematic diagram showing the dental and soft tissues angular measurements: 1) U1-PP: the angle between the long axis of the upper incisor (U1) and PP. 2) U6-PP: the angle between the PP and a line extending between U6T and U6A. 3) L1-MP: the angle between the long axis of lower incisor L1 and the mandibular plane. 4) L6-MP: the angle between the MP and a line extending between L6T and L6A. 5) Interincisal angle: the angle between the long axis of U1 and the long axis of L1. 6) Nasolabial angle: the angle between the tip of the nose (Pn), Subnasale (Sn), and Labrale superius (Ls) points. ANS = anterior nasal spine**;** PNS = posterior nasal spine; PP = palatal plane (passing between ANS and PNS)**;** U6T = tip of the mesiobuccal cusp of the upper first permanent molar**;** U6A = root apex of the mesiobuccal root of the upper first permanent molar**;** Go = Gonion**;** Me = Menton**;** MP = mandibular plane (passing between Go and Me).

**Figure 8:**
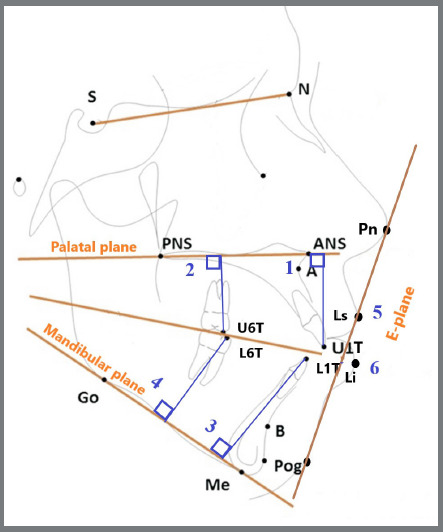
Schematic diagram showing the dental and soft tissues linear measurements: 1) U1-PP: the perpendicular distance between U1T and PP. 2) U6-PP: the perpendicular distance between U6T and PP. 3) L1-MP: the perpendicular distance between L1T and MP. 4) L6-MP: the perpendicular distance between L6T and MP. 5) Upper lip-E-plane: the distance between Ls and EP. 6) Lower lip-E-plane: the distance between Li and EP. U1T = the incisal tip of the most anterior maxillary incisor. L1T = the incisal tip of the most anterior mandibular incisor. Li = Labrale inferious. E-plane = plan passing between the tip of the nose (Pn) and soft tissue pogonion.

With the aid of WebCeph™ software, lateral cephalometric radiographs were analyzed two times, for all patients: just before the insertion of the extrusion arch (T0), and after 10 months (T1). A horizontal reference plane (palatal plane) passing between the anterior nasal spine and the posterior nasal spine was used for maxillary variables, while the mandibular plane was used as a horizontal reference plane passing between Menton (Me) and Gonion (Go) was used for mandibular variables. Then, six linear and six angular measurements were made (Figs 7, 8).

### STATISTICAL ANALYSIS

Data were analyzed using IBM-SPSS software (IBM Corp. Released 2019. IBM SPSS Statistics for Windows, version 26.0. Armonk, NY: IBM Corp). Data were initially tested for normality using Shapiro-Wilk’s test, and z-scores of skewness and kurtosis. After verifying the normality of the data, a paired-sample *t*-test was used to compare the difference between T0 and T1. Reliability was assessed using the intraclass correlation coefficient (ICC) by remeasuring digital models and cephalometric variables for 30% of the sample after three weeks. The results were considered statistically significant for p-value ≤ 0.050.

## RESULTS

This study involved 20 female patients with a mean age of 16.5±1.5 years and a mean AOB of 2.38±0.7 mm just before the extrusion arch was placed. Table 1 shows the pretreatment cephalometric data of the study sample. All twenty patients were evaluated after 10 months using the extrusion arch. Excellent intra-examiner reliability for the digital models and cephalometric measures was reported (ICC = 0.985, 0.990, respectively).

**Table 1:** Pretreatment cephalometric data of the studied sample (n=20).

Characteristic	Mean ± SD	(95% CI) (lower bound - upper bound)
SNA (degrees)	80.78 ± 1.95	(79.87 to 81.69)
SNB (degrees)	77.27 ± 1.78	(76.44 to 78.10)
ANB (degrees)	3.27 ± 1.67	(2.48 to 4.05)
SN-MP (degrees)	31.75 ± 2.02	(30.80 to 32.68)
U1-PP (degrees)	118.88 ± 3.97	(117.02 to 120.74)
LAFH (mm)	68.39 ± 3.89	(66.56 to 70.21)
Overbite (mm)	-2.38 ± 0.69	(-2.70 to -2.05)

Table 2 shows the results of the paired-sample *t*-test obtained by three-dimensional model analysis. A statistically significant increase in the amount of overbite by 4.35 ± 0.61 mm was observed. The maxillary incisors were extruded significantly by 2.52 ± 1.02 mm, with a significant increase (0.83 ± 0.2 mm) in the length of the upper incisors’ clinical crown. Significant reduction in overjet (1.58± 0.5 mm), in upper arch length (2.09 ± 0.76 mm), and in upper arch perimeter (1.9 ±0.87 mm) was observed. No significant changes in the antero-posterior position of maxillary molars and the intermolar width were observed.

**Table 2:** Mean and standard deviation (SD) of three-dimensional model measurements at T0 and T1 achieved with the skeletally-anchored extrusion arch with mean differences (T1-T0).

Measurement (mm)	Mean ± SD		MD ± SD	t (19)	p-value
	T0	T1	(95% CI)		
Overbite	-2.48 ± 0.77	1.87 ± 0.65	4.35 ± 0.61 (4.07 to 4.64)	32.047	< 0.001
Overjet	3.46 ± 0.82	1.88 ± 0.68	-1.58 ± 0.5 (-1.81 to -1.34)	-14.174	< 0.001
AP U6	7.82 ± 0.93	7.79 ± 0.95	-0.031± 0.12 (-0.089 to 0.03)	-1.084	0.292
UADH	9.1 ± 1.48	6.57 ± 0.88	-2.52 ± 1.02 (-3 to -2.05)	-11.069	< 0.001
LADH	5.86 ± 1.12	5.74 ± 1.25	-0.12 ± 0.32 (-0.27 to 0.03)	-1.713	0.103
U1 L	8.02 ± 0.6	8.85 ± 0.56	0.83 ± 0.2 (0.74 to 0.93)	18.323	< 0.001
L1 L	7.39 ± 0.6	7.4 ± 0.56	-0.002 ± 0.17 (-0.08 to 0.077)	-0.053	0.958
UAL	25.03 ± 2.02	22.94 ± 1.95	-2.09 ± 0.76 (-2.44 to -1.73)	-12.278	< 0.001
LAL	20.58 ± 1.88	20.45 ± 2	-0.13 ± 0.47 (-0.35 to 0.09)	-1.214	0.239
UAP	70.32 ± 4.39	68.42 ± 3.98	-1.9 ± 0.87 (-2.31 to -1.49)	-9.781	< 0.001
LAP	60.7 ± 3.7	60.66 ± 3.69	-0.03 ± 0.14 (-0.1 to 0.03)	-1.005	0.327
U6-6	50.03 ± 3.64	50.13 ± 3.7	0.1 ± 0.25 (-0.01 to 0.22)	1.841	0.081
L6-6	43.3 ± 2.4	43.32 ± 2.44	0.02 ± 0.07 (-0.02 to 0.05)	1.045	0.309

The test of significance used was the paired-sample t-test. SD = standard deviation. MD = Mean difference. CI = confidence interval. T0 = Pre-extrusion. T1 = Ten months post-extrusion. Statistically significant for *p*-value < 0.05.

Table 3 shows the results of the paired-sample *t*-test obtained by dental and soft tissue angular cephalometric analysis. Significant palatal tipping of the upper incisors (5.78±0.77°), and an increase in the interincisal angle and nasolabial angle (9.94±1.09°, 2.37±0.53°, respectively) were observed.

**Table 3:** Mean and standard deviation (SD) of dental and soft tissue angular cephalometric variables at T0 and T1, achieved with the skeletally-anchored extrusion arch, with the mean differences (T1-T0).

Measurement (mm)	Mean ± SD		MD ± SD	t (19)	p-value
	T0	T1	(95% CI)		
U1-PP	119.95 ± 3.78	114.17 ± 3.85	-5.78 ± 0.77 (-6.14 to -5.42)	-33.682	< 0.001
U6-PP	85.17 ± 4.22	85.15 ± 4.2	-0.01 ± 0.07 (-0.04 to 0.02)	-0.753	0.461
L1-MP	95.17 ± 4.32	94.85 ± 4.44	-0.32 ± 1.35 (-0.95 to 0.31)	-1.058	0.303
L6-MP	89.74 ± 4.2	89.72 ± 4.25	-0.02 ± 0.1 (-0.07 to 0.02)	-1.002	0.329
Interincisal angle	110.26 ± 5.38	120.19 ± 5.31	9.94 ± 1.09 (9.42 to 10.45)	40.817	< 0.001
Nasolabial angle	95.9 ± 3.8	98.3 ± 3.9	2.37 ± 0.53 (2.12 to 2.61)	20.171	< 0.001

The test of significance used was the paired-sample t-test. SD = standard deviation. MD = Mean difference. CI = confidence interval. T0 = Pre-extrusion. T1 = Ten months post-extrusion. Statistically significant for *p*-value <0.05.

Table 4 shows the results of the paired-sample *t*-test obtained by dental and soft tissue linear cephalometric analysis. There was a significant extrusion of the maxillary incisors (2.20±0.62mm) and a significant intrusion of the maxillary first permanent molars (0.02±0.03 mm), which was not clinical important. Moreover, the upper lip showed a significant tendency of retraction to the E-plane.

**Table 4:** Mean and standard deviation (SD) of dental and soft tissue linear cephalometric variables at T0 and T1, achieved with the skeletally-anchored extrusion arch, with the mean differences (T1-T0).

Measurement (mm)	Mean ± SD		MD ± SD	t (19)	p-value
	T0	T1	(95% CI)		
U1-PP	28.55 ± 2.35	30.76 ± 2.15	2.20 ± 0.62 (1.91 to 2.5)	15.933	< 0.001
U6-PP	22.96 ± 2.37	22.93 ± 2.38	-0.02 ± 0.03 (-0.04 to -0.01)	-3.286	0.004
L1-MP	41.32 ± 2.38	41.33 ± 2.37	0.006 ± 0.27 (-0.12 to 0.13)	0.100	0.922
L6-MP	29.74 ± 2.61	29.72 ± 2.61	-0.02 ± 0.08 (-0.06 to 0.02)	-1.060	0.302
UL-E plane	0.81 ± 1.8	0.69 ± 1.8	-0.12 ± 0.03 (-0.13 to -0.1)	-15.433	< 0.001
LL-E plane	1.24 ± 1.7	1.29 ± 1.6	0.04 ± 0.09 (-0.0003 to 0.09)	2.080	0.051

The test of significance used was the paired-samples *t*-test. SD = standard deviation. MD = Mean difference. CI = confidence interval. T0 = Pre-extrusion. T1 = Ten months post-extrusion. Statistically significant for *p*-value < 0.05.

## DISCUSSION

Orthodontic treatment of dentoalveolar open bite includes either anterior teeth extrusion or/posterior teeth intrusion. Many modalities have been proposed to treat AOB by extrusion of anterior teeth, such as: the multi-loop Edgewise archwire technique, maxillary accentuated and mandibular reverse-curve archwires with inter-maxillary elastics. However, most of these modalities require patient compliance and can be inconvenient. Therefore, patient compliance-independent fixed device approaches, such as extrusion arches, are increasingly being employed. Extrusion arches are an effective and predictable one-couple force system that creates a vertical extrusion force on the incisors and promotes the correction of AOB. However, the extrusion arch causes mesial tilting of the upper molars, due to the counterclockwise couple it produces. Therefore, this prospective clinical trial using a skeletally anchored NiTi extrusion arch was conducted to evaluate its effects on the anterior and posterior teeth, and soft tissues. So, habit breaking appliances (palatal crib, lingual spurs) were not used during the 10 months application period of the extrusion arch, to eliminate their effect in the correction of the AOB.^16^

For standardization and to avoid any bias in the results, patients with similar malocclusion (Table 1) were included in the present study. All the participants in this study were female adolescents (age range = 16.5±1.5 years) in the permanent dentition. The position of the extrusion arch was standardized for all patients. First, by using 0.017 × 0.025-in NiTi Connecticut intrusion archwire (Ortho Organizers, Inc.), with the same dimensions in the anterior portion (34 mm) and in the posterior portion (22 mm).^14^ The passive arch was extended from the molar auxiliary tube, while the anterior portion was incisal and in the same level to the incisor brackets, and it was tied over the segmented anterior archwire, distal to the lateral incisor brackets, delivering the same extrusive force of 30-40 g in all patients. Finally, the extrusion arch was cinched back on the distal surface of the tube of molars bands, to fix the arch length.

In this clinical trial, a skeletally anchored NiTi extrusion arch was used to treat dental AOB in adolescent patients. Skeletal anchorage was used to provide indirect anchorage for the use of the extrusion arch, and to allow for full control of posterior teeth and decrease the tip forward moment of the extrusion arch on posterior teeth. As placement site is one of the important factors for success rate and stability of the miniscrews, they were inserted in the maxilla at the mucogingival junction between the permanent first molar and the second premolar, to ensure maximum stability and avoid screws failure during treatment - as there was a great distance between their root, with good bone density -, and also to provide the maximum indirect anchorage to the posterior teeth while using the extrusion arch. Then, they were ligated around the tube of the upper first permanent molar bands, not to the hook, to avoid anchorage loss and the looseness of the ligature. Moreover, ligation of the posterior teeth and a 0.017× 0.025-in SS archwire segment was used. The use of this archwire segment in conjugation with the miniscrew would stabilize the posterior teeth, minimizing the tip forward moment of the extrusion arch on the molars.

NiTi extrusion arch was used in the study, as it delivers a force of 30-40 g^7^ on the anterior teeth, which was considered a force lower than beta-titanium (TMA) extrusion arch (which delivers a force of 40-60 g^15^) and SS extrusion arch (which delivers a force of 100 g^11^). NiTi extrusion arch was used to decrease the side effects of the extrusion arch on the maxillary molars and to get the advantages of light continuous force distribution, shape memory, and spring-back of the NiTi archwires.

In this study, an 0.017× 0.025-in NiTi extrusion arch was used as an overlay archwire, and it was tied over a 0.017× 0.025-in SS segmented main archwire, distal to the lateral incisors. The extrusion arch was used as an overlay wire, to provide a one-couple force system, and it was tied distal to the upper lateral incisor bracket over the anterior wire, with a metal ligature, to provide the proper force application point and the correct moment-to-force ratio. It was ligated over a segmental archwire, to extrude the upper incisors as one big tooth and maintain their relationship to each other.

In the present study, the results revealed that there was a statistically significant increase in the overbite by 4.35± 0.61 mm. A previous study by de Brito Vasconcelos et al^15^ achieved an increase in the overbite of 3.07±1.57 mm over 7.79 months. This difference could be explained by the longer time of the current study. On the other hand, Hammad et al^17^ reported increases in the overbite of 4.73 mm±1.93 mm. This could be explained as they used a SS extrusion arch in the upper and lower arches that exerted more extrusive force than the NiTi extrusion arch that was used only in the upper arch in the present study. 

Regarding the effect of the extrusion arch on the anterior teeth, there was a significant extrusion of 2.52 ± 1.02 mm, with clockwise development of the upper dentoalveolar process, and the length of the upper incisors clinical crown was increased significantly by 0.83± 0.2 mm. However, there was no significant change in the lower vertical development and the length of the lower central incisors. The findings for the clinical crown length were in harmony with those of de Brito Vasconcelos et al^15^ and Fouda et al^18^, who documented a significant increase in the length of incisors’ clinical crown by 0.33±0.64 mm and 0.49±0.63 mm, respectively. This increase can be explained by the fact that, during extrusion of the incisors, the gingiva may not conform to the tooth, which may increase the clinical crown height.

Regarding extrusion of the anterior teeth, these findings agree with those of Hammad et al^17^ and Erdem and Kucukkeles^19^, who reported significant extrusion of the upper incisors by 2.05±0.72 mm and 2.16 mm, respectively. However, they also reported significant extrusion of the lower incisors, by 2.54±1.63 mm and 1.49 mm, respectively - these results were not consistent with the present study. This can be attributed to only using the extrusion arch on the upper arch in the current study, while the lower arch was stabilized during extrusion - on the other, hand Hammad et al^17^ used the extrusion arch in the upper and lower arches. In addition, Erdem and Kucukkeles^19^ used maxillary accentuated and mandibular reverse-curve archwires with inter-maxillary elastics.

The results of this study revealed that there was significant palatal tipping of the maxillary incisors by -5.78±0.77°, and there was no significant change in the inclination of the lower incisors. These results can be explained as there was an uprighting moment produced by the extrusion arch on the upper incisors,^11^ effect that is favorable in patients with labial inclination of the maxillary incisors. Moreover, this study showed that the interincisal angle increased significantly by 9.94±1.09°, due to the palatal tipping of the anterior teeth. These results were consistent with those of Hammad et al,^17^ Atsawasuwan et al,^20^ and de Brito Vasconcelos et al.^15^ As a result of incisor palatal tipping, the overjet reduced significantly by 1.58± 0.5 mm. These results were consistent with those of Atout et al,^21^ Kim et al,^8^ de Brito Vasconcelos et al,^15^ and Kraisiridej et al.^22^

Regarding the effect of the extrusion arch on dental arches parameters, the results of the current study showed that the upper arch length and the upper arch perimeter reduced significantly by 2.09 ± 0.76 mm and 1.9 ±0.87 mm, respectively, with no significant change in the lower arch length and perimeter. These results were similar, but they were greater in magnitude, to those of Aliaga-Del Castillo et al^23^, who used bonded spurs, and Slaviero et al^24^, who used fixed and removable palatal cribs. This can be explained as in this study the extrusion arch exerted a force located anterior to the center of the resistance of the anterior teeth, resulting in a moment tending to tip the incisors palatally; while the tongue spurs or crib depends on change in the muscle equilibrium, tending to pure extrusion of the upper incisors. In the present study, there were no significant changes in the intermolar width. This finding was consistent with those of de Brito Vasconcelos et al.^15^

Regarding the effect of the extrusion arch on the upper permanent molars, there was no statistically significant difference in their anteroposterior position (p>0.005). A randomized clinical trial conducted by Fouda et al^18^ showed that there was a significant mesial tipping of the upper first molar by 1.42 ±0.99 mm and 0.53±0.32 mm in the conventional fixed palatal crib group and the miniscrew-supported palatal crib group respectively. This difference can be attributed to using different treatment techniques, as they used palatal crib and inter-tooth space in mixed dentition that may result in a more mesial movement of the molars, while in the current study skeletally anchored extrusion arch was used in patients with permanent dentition.

The maxillary first permanent molars were intruded significantly by 0.02±0.03mm, but this amount of intrusion is considered clinically ineffective, and there was no change in the vertical position of the lower first permanent molars. These findings were less than the results reported by Hammad et al,^17^ Kim et al,^8^ and He et al^25^, who revealed significant intrusion of the maxillary first permanent molars by 0.95mm, 0.66mm, and 0.4mm, respectively. The difference in the amount of intrusion can be explained by using mini-screws as indirect anchorage to minimize the moment produced by the extrusion arch on the molars, because of the one-couple force system.

Regarding maxillary and mandibular molars inclination, this study found no statistically significant difference between before and after extrusion. These findings were in contrast with those of de Brito Vasconcelos et al^15^, who revealed significant maxillary molar mesial tipping of 11.49± 8.41°. This difference can be explained as in that study miniscrews were used as indirect anchorage to decrease the side effect of extrusion arch on the molars, and the buccal segments were included and ligated as additional anchorage, using 0.017× 0.025-in SS archwire - while de Brito Vasconcelos et al^15^ used extrusion arch only as overlay wire the over 0.014 × 0.025-in CuNiTi archwire, without including the buccal segments. 

Regarding soft tissue changes, the results of this study indicated that the upper lip showed a significant retraction tendency toward the E-plane, without a significant change in the position of the lower lip, with a significant increase in the nasolabial angle. These results coincide with the results of Cozza et al^26^ and Giuntin et al^27^. These results can be attributed to palatal tipping of the upper incisors, leading to retrusion of the upper lip to the E-plane and the increase in the nasolabial angle.

## CONCLUSIONS

» Skeletally anchored NiTi extrusion arch was an effective technique for treating AOB, with no adverse effects on the molars.
» A mean overbite correction of 4.35 ± 0.61mm was produced by the skeletally anchored NiTi extrusion arch over the ten-month follow-up period.
» Palatal tipping and extrusion of maxillary incisors, with a reduction in upper arch length and perimeter, and retraction of the upper lip was associated with the use of the extrusion arch.
» A statistically significant intrusion of the maxillary first molars occurred due to the moment of the one-couple force system; however, with minor clinical significance.
